# ‘Being a Good Parent’ for Seriously Ill Children in Brazil: A Qualitative Semantic Analysis

**DOI:** 10.1111/nicc.70149

**Published:** 2025-08-11

**Authors:** Fernanda Machado Silva‐Rodrigues, Pamela S. Hinds, Maiara Rodrigues dos Santos, Lucas Thiago Pereira da Silva, Regina Szylit

**Affiliations:** ^1^ School of Nursing University of São Paulo (USP) São Paulo Brazil; ^2^ Department of Nursing Science, Professional Practice and Quality, Children's National Health System, Professor of Pediatrics The George Washington University Washington DC USA

**Keywords:** childcare, critical care, parenting, patient care planning, qualitative study

## Abstract

**Background:**

Most studies on ‘being a good parent’ focus on North American populations, leaving a gap in understanding parents' cultural beliefs and definitions in other regions. Cultural values, societal norms and personal experiences shape these beliefs, influencing their caregiving roles and actions.

**Aims:**

This qualitative study explored Brazilian parents' beliefs about being ‘good parents’ to seriously ill children in the paediatric intensive care unit (PICU) and examined parents' descriptions of healthcare professionals' behaviours that support them in this role.

**Study Design:**

The study used a convenience sample of 15 parents of 15 children diagnosed with life‐threatening clinical conditions. Semi‐structured interviews were conducted and analysed using inductive semantic content analysis.

**Results:**

The analysis revealed four primary themes: ‘Being present for my child’ (*n* = 12, 80%), where parents highlighted the need for continuous physical and emotional availability; ‘Providing protection’ (*n* = 5, 33%), emphasising their role in safeguarding their child's well‐being; ‘Maintaining emotional balance and patience’ (*n* = 2, 13%), which reflected the emotional regulation needed to navigate the PICU experience; and ‘Effective communication with healthcare professionals’ (*n* = 2, 13%), underscoring the importance of clear information and collaborative decision‐making. These themes reflect core parental values, including unwavering support, ensuring their child's safety and maintaining a sense of control in the uncertain environment of the PICU.

**Conclusions:**

The study reveals Brazilian parents' beliefs about being a ‘good parent’ in the PICU, emphasising the importance of parental presence, specific behaviours and family unity in ensuring their child's well‐being. It also highlights the role of effective communication and information from healthcare professionals, which allows parents to make informed decisions and engage actively in their child's care.

**Relevance to Clinical Practice:**

Understanding Brazilian parents' cultural beliefs and expectations underscores the need for a comprehensive approach to caregiving in the PICU, where critical care nurses play a key role. By providing emotional support, clear communication and guidance, nurses can help parents navigate their caregiving role, reducing stress and enhancing parental involvement. Tailoring communication and support strategies to these needs can strengthen family‐centred care and improve the overall experience for parents and their seriously ill children.

**Impact Statements**

- What is known about the topic
  ○ Most research on ‘being a good parent’ in caring for seriously ill children has focused on North American populations.
  ○ Cultural values, societal norms and personal experiences shape parents' beliefs about being ‘good parents’, influencing their caregiving roles and actions.
  ○ Family‐centred care in paediatric intensive care units (PICUs) involves recognising parents as partners in decision‐making, whose psychological and emotional well‐being is crucial to the caring process.
- What this paper adds
  ○ This study provides insights into Brazilian parents' beliefs about being ‘good parents’ to seriously ill children in the PICU, emphasising the importance of parental presence, specific behavioural attributes and family unity.
  ○ It identifies behaviours of healthcare professionals that support parents in fulfilling their caregiving roles, such as vigilance, clear communication and compassion.
  ○ The findings underscore the need for tailored communication and support strategies that align with Brazilian cultural beliefs and expectations, enhancing parental involvement and the overall care experience for seriously ill children.

## 1 Introduction

In recent years, the notion of ‘being a good parent’ has gained prominence in research, especially in the context of parents caring for ill children [1, 2, 3]. Researchers have noted that parents often align their personal beliefs and priorities with their intrinsic understanding of being a good parent [1, 2, 3, 4, 5]. This trend has evolved over a decade, marked by observations of parents integrating their unique beliefs into their caregiving roles, emphasising the importance of caring and being attentive to their children's needs [2, 5].

Despite the significant body of literature already produced on the subject, there are gaps in understanding the definition of ‘being a good father/mother’. To address this gap, this study specifically explored the beliefs of Brazilian parents about being a ‘good parent’ to seriously ill children hospitalised in a paediatric intensive care unit (PICU) and examined parents' descriptions of healthcare professionals' behaviours that support them to perform their roles.

## 2 Background

The primary causes of hospitalisation among children in PICUs in Brazil can vary over time and across different regions. Across the paediatric lifespan, the leading cause of hospitalisation in Brazilian PICUs is respiratory illness, followed by congenital cardiac illness [6].

A critically ill child exhibits severe respiratory and circulatory disorders, along with acute deterioration of consciousness. The original term ‘seriously ill child’ is used to refer to children with life‐threatening clinical conditions often associated with a poor prognosis, including the potential for death or significant long‐term consequences [3]. Regarding taking care of a seriously ill child, parents may have different beliefs and perspectives about the meaning of being a good parent. These beliefs can shape their decisions and actions regarding advance care planning for their children [2, 4, 5].

Studies emphasise the importance of family‐centred care in PICUs [7, 8]. This approach involves acknowledging parents' unique and invaluable perspective in caring for their critically ill children. Family‐centred care recognises that parents are not just caregivers but key partners in decision‐making processes. Parents' psychological and emotional well‐being is paramount, as their ability to cope with the stress of hospitalisation directly impacts their decision‐making capabilities and interactions with healthcare professionals. Family‐centred care aims to provide support that respects and addresses the diverse needs and preferences of families, ensuring that their voices are heard and valued in the care of their child [7].

The literature also sheds light on parental personal sense of duty as a foundational aspect of paediatric medical decision‐making [9, 10]. Parents often find themselves in the difficult position of balancing their child's immediate medical needs with long‐term outcomes and quality‐of‐life considerations. This balance is particularly challenging in life‐threatening conditions common in PICUs [2, 10]. A sense of duty influences how parents prioritise various treatment options, often placing a significant emotional burden on them. Recognising and supporting this personal sense of duty is key for healthcare providers to facilitate more effective and compassionate communication with families, leading to decisions that are in the child's best interests and align with the family's values and expectations [5, 9].

Having a child hospitalised in a PICU is a significantly stressful experience for parents [10]. Upon the diagnosis of a severe illness in their child, in addition to the emotional repercussions and changes in routine and family dynamics, these parents will face various decisions related to their children's healthcare [10, 11]. In this regard, researchers believe that parents base such decisions on several factors, including medical recommendations from healthcare professionals, individual and cultural values, beliefs about what they perceive as most urgent for the child's well‐being, hopes for clinical improvement, personal beliefs and internal definitions of what constitutes being a good parent in adverse clinical circumstances [9, 11].

### 2.1 Aims

To explore the beliefs of Brazilian parents about being a ‘good parent’ to seriously ill children hospitalised in a PICU and to examine parents' description of healthcare professionals' behaviours that support them to perform their roles.

## 3 Design and Methods

### 3.1 Study Design

A descriptive qualitative approach through inductive semantic content analysis [12] guided the analysis of parental beliefs about ‘being a good parent’ for a seriously ill child. Inductive semantic content analysis was selected for its proven effectiveness in identifying, counting and interpreting the specific terms used by participants. This method enables the capture of nuanced and implicit connotations in their narratives and has been previously employed in similar studies [13, 14].

While other forms of content analysis, such as thematic analysis, offer a deeper dive into the meaning of underlying themes and patterns within the data, our focus was primarily on the parents' explicit language and direct expressions. Our analysis was guided not by the intention to generate a new theoretical construct, but rather to deepen the understanding of the existing concept of ‘being a good parent’ within a specific cultural and clinical context, allowing us to capture both manifest content (explicit terms used) and latent meanings (underlying values and beliefs). The report followed the recommendation of the consolidated criteria for reporting qualitative studies (COREQ) [15].

### 3.2 Setting and Sample

A convenience sample comprising parents of seriously ill children, all of whom had been hospitalised in a PICU, was used to ensure variation in demographics and clinical conditions. The inclusion criteria were parents older than 18 years who had a child hospitalised in the PICU at the time of the study's data collection and parents directly involved in the care of their hospitalised child. We excluded parents experiencing heightened emotional vulnerability, as identified and indicated by the healthcare team. Every parent invited to participate in the study agreed, except for one mother whose child experienced clinical instability on the day the interview was scheduled. Following the principles of qualitative research, specifically semantic content analysis, the sample size was not predetermined, as this approach relies on saturation at the code level to ensure comprehensive data representation [16].

### 3.3 Data Collection

Semi‐structured interviews were conducted using a standard interview format, as in previous studies about good parental definitions [1, 5, 15, 17, 18, 19]. The interviews were conducted in a public hospital specialised in the care of children with complex conditions, which serves patients from diverse socio‐economic backgrounds and regions of Brazil. Two nurses conducted the interviews. One was a novice nurse who was new to qualitative research and conducted the interviews under the guidance of the second nurse, who holds a PhD and is an experienced qualitative researcher.

Parents were first asked to report the general aspects of their children's illnesses and what caused them to be hospitalised. The author's definition of ‘being a good parent’ of a very ill child was then approached. Participants were also asked about aspects of their relationship with the clinicians that would help them achieve their good parental definition. Data collection continued until saturation was reached at the code level [20], ensuring a comprehensive and representative coding scheme. Saturation was confirmed when the dataset was sufficiently extensive to capture the full range of parental perspectives [16, 20].

Parents' responses were recorded, transcribed and read to the participants the following day of the interview for content verification. During the interviews, the interviewer also used clarifying prompts (e.g., ‘May I confirm your meaning with that important response?’) to ensure accurate understanding and interpretation of participants' statements. This technique provided real‐time validation of participants' meanings without requiring follow‐up contact. Parent and child demographic variables were also collected.

### 3.4 Data Analysis

The transcribed responses were analysed via semantic content analysis [12] with the support of the software Atlas.ti 23 [21]. Per the procedures described by Krippendorff, (1) each response to each interview question was the unit of response and each sentence was the unit of analysis and was assigned a code based on the precise and recurring language used by parents, which captured their intended meaning and, consequently, the meaning of the codes; (2) each uniquely encoded sentence underwent thorough comparisons with real quotes from the parents' interviews and with all other encoded sentences to guarantee the accurate representation of the parents' intended meaning and to evaluate the similarities or expanded interpretations among recurring codes; (3) a conceptual definition was created for each code, drawing from the specific language used by the parents and the interpreted meaning as evaluated by them; and (4) the recurring and similar codes were merged to minimise the number of distinct codes. This merging process led to a revision of the code definitions, encompassing all the elements of the combined codes, which were then designated as themes (also described as factors) along with their respective definitions. Disagreements in the perspectives of codes or themes were resolved through discussion and consensus. (5) The various components of each theme were compared and extracted to create a comprehensive conceptual definition of what it means to be a good parent.

### 3.5 Methodological Rigour

Regarding methodological rigour, confirmability was ensured by involving multiple investigators in data collection and analysis, supported by dialogic and reflective discussions. The continuous, collaborative and multifaceted examination of data among researchers fostered reflexivity throughout the research process, enabling consensus and enriching the analysis while embracing the researchers' subjectivity. Credibility was established through prolonged engagement in the study setting, leveraging the researchers' expertise in qualitative interviews and conducting regular debriefing meetings throughout the research process. The transferability of the study findings was enhanced by thoroughly discussing data saturation at the code level, providing a detailed description of the research process, establishing an audit trail and ensuring dependability. Additionally, our coding scheme was based on established validation criteria described by Krippendorff: face validity, social validity and empirical validity.

### 3.6 Ethical Considerations

Ethical approval was granted by the Institutional Review Board of Santa Casa de São Paulo Hospital, São Paulo, Brazil (5.339.610/2022). Informed consent was obtained from all parents included in this study.

## 4 Results

Fifteen parents of 15 children hospitalised in the PICU, 13 mothers and 2 fathers participated in this study. Most parents were mothers (86.7%), and the parents' ages ranged from 21 to 41 years. All parents (*n* = 15) defined themselves as primary caregivers. Most patients were male (60%), and their ages ranged from 4 months to 14 years. Respiratory diagnoses were the most common (*n* = 6; 40%) (Table 1).

**TABLE 1:** Sociodemographic and clinical characteristics of paediatric patients and their primary caregivers.

	Primary caregiver (*N* = 15)		
	No.		%
Mother	13		86.7
Father	2		13.3
Age (years)			
Parent			
Mean		30.8	
SD		7.0	
Median		29	
Range (min–max)		21–41	
Patient			
Mean		2.9	
SD		3.9	
Median		1.1	
Range (min–max)		4 months–14 years	
Sex (child)			
Male	9		60
Female	6		40

Primary diagnosis category	No.	%
Oncologic	1	6.7
Respiratory	6	40
Gastrointestinal	1	6.7
Metabolic/genetic	4	26.5
Skin/muscles	1	6.7
Cardiac disease	1	6.7
Chronic kidney disease	1	6.7

We interviewed each mother or father once; the average interview duration was 29 min. All the interviews occurred in a private room inside the PICU. All parents were interviewed individually, as just one parent participated for each child. To preserve participants' identity and ensure anonymity, each participant was assigned a numerical code ranging from 1 to 15.

According to the analysis employed, our study identified explicit expressions parents used to define ‘being a good parent’ while also considering the underlying cultural and emotional values these definitions reflect. By focusing on the precise language parents use, we identified recurring patterns and meanings that reflected their caregiving beliefs and motivations. Our findings were organised by interview questions, with corresponding tables displaying the frequency of codes, following the approach used in previous studies that employed the same methodology [12, 22].

*Analysis of Question 1. Please, in your own words, tell me what* ‘being a good father/being a good mother’ *means to you, considering the current clinical condition of your child*.

Table 2 presents the codes, their definitions, the number and frequency of parents endorsing each code and sample quotes. Most parents (80%, *n* = 12) endorsed presence as the main code, allocated to the theme ‘Being present for my child’. Presence was defined as ‘Parents want to be close to their child; spend quality time together/provide continuous support, protection, and active participation in the child's life and care’. Additionally, 33.3% (*n* = 5) of parents mentioned protection, 13.3% (*n* = 2) mentioned support and 13.3% (*n* = 2) mentioned actively participating in care.

**TABLE 2:** Themes and codes related to being a good parent of a seriously ill child.

			Codes *N* = 55		Parents reporting *N* = 15		
Theme	Codes	Definition	No.	%	No.	%	Sample quotes
Being present for my child	Presence	Parents want to be close to their child; spend quality time together; provide continuous support, protection and active participation in the child's life and care	15	27.4	12	80	‘In my opinion, being a good mother means being as close as possible to your child’. P1 ‘I believe that being a good father or mother means being present, having a quality presence’. P7
	Protection		9	16.4	5	33.3	‘To me, being a good mother means to protect. I felt that with my son; I want to protect him’. P 3
	Support		2	3.6	2	13.3	‘Therefore, I say: Calm down, I'm going to hold your little hand, and everything will be all right because I am here with you’. P 15
	Actively participate in the care		2	3.6	2	13.3	‘I also do my part in taking care of him. I don't let the nursing staff do everything. Whatever I can do and manage, I am doing it’. P13
Acting like a good mother/a good father	Show emotional balance	Parents recognise the need to control their feelings and reactions; inform themselves about the child; show strength and attention and to communicate, entertain and play with the child	2	3.6	2	13.3	‘Talking to him without showing too much despair. Some mothers cannot hold back their emotions, but if you don't control yourself, you end up not helping and scaring the child even more’. P1
	Be patient		2	3.6	2	13.3	‘To have patience with him and the situation, without mistreating him. In addition, without mistreating the people here taking care of him’. P2
	Communicate with the child		2	3.6	2	13.3	‘I talked to him, telling him that it was to make him feel better, to help him, and that his family and I were waiting for him at home’. P1
	Search for information		2	3.6	2	13.3	‘Now, what I do is I am even more communicative, paying close attention. Whenever I don't understand something, I search for it online to see what is happening and what procedures are being done. Therefore, I am being a good mother by performing this role’. P4
	Entertain and play		2	3.6	2	13.3	‘I want him to feel that he is not alone, that he has a mother who will protect him, talk to him, and play games just to see him a little less sad’. P5

	Be attentive		2	3.6	2	13.3	‘Being a good mother is paying close attention to my child, much more than before. Not neglecting him or thinking that any issues will just pass. I believe that is it, paying more attention to my child’. P9
Be strong	2		3.6	2	13.3	‘To offer and be the strength she needs during this incredibly difficult time she is going through’. P9
Supporting the family	Promote family union	Parents believe it is their duty as a good parent to keep the family united and to embed certain principles, beliefs or moral standards in the child's upbringing	2	3.6	4	26.7	‘To keep my family united and present, me, my husband, and my other child, to provide support for (child) and ourselves as well, to keep us strong and present’. P2
	Sustaining the family		3	5.6	2	13.3	‘My entire family and I, knowing how to deal with the situation and supporting each other by staying united, this is the foundation of everything’. P14
	Instil family values in the child		2	3.6	1	6.6	‘Each person treats their child as they believe to be a good parent, which stems from a family upbringing. For instance, how you were raised and what you consider as a good mother, you will pass on to your child’. P2
Experiencing frustration	Guilty	Parents experience negative emotions and self‐disappointment as they strive to be the best parents, they can be	4	7.4	2	13.3	‘Whatever I do, it never seems enough, as if it is our fault, you know?’ P5
	Powerless		2	3.6	2	13.3	‘As parents here in the hospital, we feel so incapable, especially when we are not from the medical field and don't understand anything. It is like we are useless’. P6

The second theme identified by grouping the codes obtained was ‘Acting like a good mother/a good father’. In this theme, a balance was observed in the codes endorsed by parents. Each code, including ‘show emotional balance’, ‘be patient’, ‘communicating with the child’, ‘searching for information’, ‘entertain and play’, ‘be attentive and ‘be strong’, was endorsed by 13.3% (*n* = 2) of parents’.

Another theme was ‘Supporting the family’, in which ‘promoting family union’ was cited by 26.7% (*n* = 4) of the parents. According to their definition, the participants believe that ‘it is their duty as a good parent to keep the family united and to embed certain principles, beliefs, or moral standards in the child's upbringing’.

The last theme, ‘Experience frustration’, encompassed the codes ‘guilty’ and ‘powerless’. According to these codes, 13.3% (*n* = 2) of parents experience negative emotions and self‐disappointment as they strive to be the best parents they can be.

*Analysis of Question 2. It is important for the team caring for your child to do everything within their reach to support your definition of* ‘being a good father/being a good mother’. *Please describe to me what the professionals caring for your child could do to help you better fulfil your role as* ‘a good father/a good mother’?

Regarding the contributions of the clinicians in supporting parents in fulfilling their role as good parents, Table 3 shows the themes obtained, the codes and their definitions, as well as the frequency of codes and parents reporting each code.

**TABLE 3:** Health professionals' contributions to supporting parents in fulfilling their role as good parents.

			Codes *N* = 49		Parents reporting *N* = 15		
Theme	Codes	Definition	No.	%	No.	%	Sample quotes
Ensuring that they are vigilant and attentive	Awareness	Being vigilant and attentive means being watchful, alert and focused, with a heightened sense of awareness, care and attention to ensure the well‐being, safety and quality of care provided.	3	6.1	5	33.3	‘They take care of him (child), watch over him, stay close to him, make sure everything is alright. They check if he's not feeling sick or if something serious is happening, give him the medication. I already find that quite difficult. In addition to caring for a human being who is almost leaving, must be very hard’. P1 ‘They are always keeping an eye on him (child), to the point that I can rest assured knowing they are watching over him, so I don't have to worry about that. They do everything they can, even the impossible’. P12
	Careful		2	4.1	2	13.3	‘Therefore, everyone here is awesome, always caring. Asking all the time how he's doing, making sure everything is going well, giving him his medications, being truly careful’. P3
	Attention		2	4.1	2	13.3	‘They're always genuinely concerned. Asking all the time how he (the child) is doing and if everything is truly going well’. P3 ‘In the PICU, the professionals are excellent, so continuing to care for him with attention and individuality is enough for me’. P14
Providing information	Information	Providing information involves sharing accurate details, fostering transparency, addressing divergences and promoting effective communication for better understanding and collaborative decision‐making.	11	22.4	6	12.2	‘From what I see here, for the second time, they truly take good care, informing and giving advice’. P1 ‘Getting information from the team helps to understand what's happening there. Whether the child can get better or worse’ P1
	Divergences		1	2	1	6.6	‘Avoiding disagreements of opinions among the professionals because that just confuses us.’ P13

Transparency		2	4.1	2	13.3	‘My daughter started vomiting a lot, and we got worried. We asked what was happening, but the professionals couldn't explain why. It is truly distressing because we didn't study this, we don't have that knowledge. It's hard to deal with this uncertainty. I wonder why I'm here if I can't know what's going on and they don't have a solution. It is important to have better communication to help and make decisions together’. P4
Being flexible and comprehensive	Flexibility	Being kind and compassionate means being adaptable, understanding and open‐minded in order to accommodate different perspectives and adjust plans when necessary. It involves being inclusive and considering individual circumstances.	2	4.1	2	13.3	‘I think it's for the professional not to be arrogant, and for us as well. Both sides trying to understand each other already helps a lot. Having that flexibility, knowing how to adapt when needed, to help us face something or get out of difficulties makes the situation flow’. P2
	Comprehensiveness		3	6.1	2	13.3	‘Just one thing I have to add, a single complaint, would be for the hospital professionals to have more understanding when we arrive because I live far away, and they expect me to be here at a certain time, but I'm from the coast. I'm often on the other side of the world taking care of my other children’. P15
Being kind and compassionate	Humanity	Being kind and compassionate means showing genuine care, empathy and understanding towards others and treating them with affection, respect and empathy. It involves combining technical proficiency with a heartfelt concern for the well‐being of others.	1	2	1	6.6	‘However, I think there's still a bit of a lack, both in the medical and nursing side, of being more human in the process, you know? Not that the technical part is not important, but I think it needs to combine the technique with humanity’. P7
	Kindness		2	4.1	1	6.6	‘Therefore, I truly like the team, most of them are truly caring, you know? They treat him (the child) with a lot of affection’. P5
	Empathy		2		1		‘They treat us well and with empathy’. P2
Caring for the child and the family	Being present	Caring for the child and the family involves being present, promoting participation and valuing the contributions of healthcare professionals and family members. It creates a supportive environment that fosters learning and collaboration for the child's well‐being.	1		1		‘So, that's it. Having love and care for the child and the family. Being present in our moment is important and makes everything less heavy and complicated’. P3
	Promote participation		4		3		‘In some activities, we are included as well. This is very important. Maybe involving the father and mother during a specific non‐invasive procedure. I know it's not everything, but we want to be useful, we want to be parents. Being together, side by side with our child is so important for us and them too. Ultimately, it is about participating in the day‐to‐day’. P7 ‘They don't invalidate me; they let me be her mother and stay with her; that's what matters most’. P4
	Educate and learn		2		2		‘They offer me the opportunity to participate in my child's treatment process. Allowing me to learn from them about the best way to take care of him makes me feel useful’. P13 ‘…we learn from them, and they learn from us.’ P2
Leaving positive impressions	Positive mark	Leaving positive impressions means making a meaningful impact on others that enriches their lives. It involves creating lasting, favourable memories and experiences.	1		1		‘They help me a lot; we become familiar with the team over time. Every professional who comes here leaves a positive mark on my life’. P10

In the first theme, ‘Ensuring that they are vigilant and attentive’, one of the prominent codes identified was ‘awareness’, 6.1% (*n* = 3), which highlighted the importance of professionals being vigilant and attentive. This code, defined as being *watchful, alert and focused on ensuring the well‐being and safety of the child*, was endorsed by some parents, indicating its critical role in the caregiving environment.

Another theme was ‘Providing information’, where parents emphasised the need for clear and accurate communication from healthcare professionals. This theme includes codes such as ‘information’ and ‘transparency’, which involve sharing detailed, straightforward information to foster better understanding and collaborative decision‐making.

The theme ‘Being kind and compassionate’ reflected the need for healthcare providers to be adaptable and understand each family's unique circumstances. This includes being flexible and adjusting plans to accommodate parents' different perspectives and requirements. Lastly, the theme ‘Being kind and compassionate’ captured the desire of healthcare professionals to combine technical proficiency with genuine care and empathy. This theme is pivotal in ensuring the caregiving environment is clinically efficient while demonstrating genuine care, empathy and emotional support.

These themes collectively depict the multifaceted role that healthcare professionals play in aiding parents to navigate their duties under the challenging circumstances of having a seriously ill child.

## 5 Discussion

The objective of this study was to examine the beliefs of Brazilian parents regarding what it means to be a ‘good parent’ for seriously ill children hospitalised in the PICU. The findings provide insights into the perspectives and cultural beliefs surrounding parental roles in the context of serious childhood illness.

One key finding of this study was the emphasis parents placed on the importance of being present for their seriously ill child, emotionally and physically, despite this being more feasible for some than for others. Even so, parents expressed their wish to remain actively involved, providing continuous support, protection and care throughout the hospitalisation. This commitment to presence aligns with previous research showing that parental presence and involvement can positively impact the child's emotional and physical recovery [3, 4, 11, 15, 23, 24].

The strong emphasis on presence suggested that parents perceive their physical and emotional availability as fundamental to their identity as caregivers. In a high‐stress environment like the PICU, presence served as both a reassurance to the child and a means for parents to reaffirm their role, providing them with a sense of purpose and control in an otherwise uncertain medical setting. Like other studies conducted in the United States and China [1, 11, 23, 25], our results underscore how, in moments of vulnerability, presence is not only about physical proximity but also about the fulfilment of parents' emotional and symbolic role as caregivers.

These results are also consistent with family‐centred care literature, which emphasises the importance of parental involvement in paediatric intensive care [24, 26]. At the site of this study, only one parent was allowed to accompany the child during their stay in the intensive care unit. More broadly, within the Brazilian context, hospital policies and resource limitations can further restrict the possibility of both parents being physically present at their child's bedside [2, 24, 27]. In addition, many parents face conflicting demands between work and hospital schedules and institutional policies that prohibit overnight stays. Being mindful of these challenges is key to developing hospital policies that facilitate parental involvement and better shape their definitions of ‘being a good parent’.

Additionally, parents emphasised certain behavioural attributes and qualities associated with being ‘good parents’ in the PICU. These included displaying emotional balance, patience, effective communication with the child, seeking information, engaging in activities that involve entertainment and play, and being attentive. In combination, these traits reflected an implicit belief that ‘good parents’ demonstrate emotional resilience, cognitive engagement and active participation in the child's medical journey [1, 4, 28]. Emotional balance and patience highlight parents' awareness of their influence on their child's coping mechanisms. The urge to ‘search for information’ stems from a need to regain control over their child's care and affirm their role as informed and responsible caregivers. Moreover, parents used the information to assert their voice in decision‐making, a role often unclear or discouraged in the Brazilian healthcare setting [27]. These attributes align with parents' broader understanding of their role in providing emotional support, fostering a positive environment and actively participating in their child's care, as documented in the literature [3, 17, 23, 29].

A recent Chinese study on the concept of being a good parent in the context of childhood cancer revealed that Chinese parents prefer to express love through actions rather than words [25]. In contrast, in the Brazilian context, parents tend to balance feelings and actions.

It is noteworthy to address the observed diversity in the codes/themes reported in Table 2, where many of these codes/themes have low frequencies in comparison with Table 3. This difference in the number of parents reporting each code/theme might highlight the uniqueness of these definitions. The lower frequency of codes/themes, with fewer than one to three parents reporting them, suggests that these definitions are highly individualised. Consequently, this emphasises the need for clinicians to assist parents in fully expressing all aspects of their internal definitions. By understanding and acknowledging the unique perspectives of each parent, clinicians can better support them and address their specific needs and concerns.

Furthermore, our findings highlighted the importance of family unity in the beliefs of Brazilian parents. Parents expressed a sense of responsibility in promoting family cohesion and instilling certain principles, beliefs or moral standards in their child's upbringing. This underscores parents' recognition of the broader family context and their role in shaping their child's values and well‐being. For families of seriously ill children, the relationship with healthcare providers is also an essential component in helping parents manage the situation, supporting both the ongoing survival and sustenance of family relationships [26].

The ‘supporting the family’ theme illustrated the collectivist nature of Brazilian family dynamics, where caregiving is not an isolated parental duty but an interconnected responsibility involving the entire family. This reinforces the belief that parental success is measured not only by the child's care but also by the well‐being of the broader family unit [26, 30]. This suggests that, in the Brazilian context, parental identity is closely tied to their ability to uphold family cohesion, even in the face of medical adversity.

Our findings can be further interpreted through the Communication Theory of Identity (CTI), which conceptualises identity as emerging through four interrelated frames: personal (self‐definition), enacted (expressed through behaviours and decisions), relational (shaped through interactions) and communal (influenced by cultural and social norms) [31]. Brazilian parents' emphasis on being present and protective corresponds with the personal and enacted frames, while their focus on family unity and shared values reflects the relational and communal dimensions. This multifaceted view of identity construction aligns with recent studies that applied CTI to examine how parents of seriously ill children define and perform their ‘good parent’ role [2, 4]. In these studies, CTI helped illuminate how communication with healthcare professionals can affirm or challenge parental identity, especially during emotionally complex decisions or transitions in care.

The frequent mention of ‘protection’ highlights a deeply embedded cultural expectation of parental duty, where shielding one's child from harm, whether physical, emotional or procedural, is seen as a primary responsibility. For many parents, actively participating in their child's care is an extension of this protective instinct, symbolising an assertion of agency in a setting where they often feel powerless. This reinforces the idea that Brazilian parents strive to influence their child's well‐being in high‐stakes medical situations, even when formal medical decision‐making is culturally limited.

The findings show the parents' commitment to checking their child's health, providing necessary medication, addressing potential concerns and being vigilant and attentive as caregivers. By comparing the Brazilian parents' definitions with the literature, we can observe the consistent importance of vigilance and attentiveness in parenting [1, 7, 16, 27]. The quotes highlight the parents' dedication and commitment to closely monitoring their children's well‐being, ensuring their safety and going above and beyond to meet their needs. These findings contribute to our understanding of the universal nature of these parental definitions and their relevance across different cultural contexts.

The study also analysed definitions related to the contributions of professionals in helping parents fulfil their role as ‘good parents’. Providing information emerged as a key factor, empowering parents to understand their child's condition, make informed decisions and actively participate in their care. Quotes underscored the significance of accurate, timely information from healthcare professionals in shaping parents' understanding, decision‐making and overall experience [3, 5, 8, 11].

It is important to connect parents' definition of ‘Being present for my child’ with how healthcare providers can support this presence. Healthcare providers should primarily be flexible and accommodating, considering circumstances such as the distance from the hospital and parental availability. Treating parents with kindness, compassion and respect fosters a supportive environment, easing emotional stress in the context of uncertainty. Additionally, encouraging parent participation in care activities can help parents feel more involved and confident in their caregiving role.

Parents appreciated the professionals' efforts in keeping them informed and guiding them in their caregiving role. The findings related to the provision of information reflected the parents' desire to be well informed about their child's health status. They highlighted the importance of communication in managing expectations and uncertainties [32, 33].

Our results indicate that parents see healthcare professionals as extensions of their protective role, relying on them for vigilance in their absence. Likewise, their emphasis on kindness and compassion suggests they value technical expertise and emotional sensitivity. Similar findings confirm that parental expectations extend beyond clinical competence, including empathy and emotional support [2, 30, 34, 35].

Healthcare professionals are more likely to empower parents, particularly in stressful settings like the PICU, where uncertainty is overwhelming. When hierarchical medical structures limit family involvement in decision‐making, such as in Brazilian healthcare, parents may need more explicit communication to determine their position in their child's care. Similar studies conducted in North America found that shared decision‐making can reduce parental stress [7, 23, 30, 36, 37, 38]. However, where Brazilian culture prevails, cultural expectations may influence how parents interact with healthcare teams. Therefore, ensuring that the communication strategies are adjusted to accommodate these cultural expectations can enhance family‐centred care.

Some studies emphasise the importance of effective communication and information sharing between healthcare professionals and parents [4, 27, 33]. According to other parents' definitions, good parents are those who are engaged, informed and able to make decisions based on accurate and timely information. Our findings align with these findings, as they demonstrate the parents' desire for information, their need to understand their child's condition and their expectations for better communication with the healthcare team.

The quotes emphasise the importance of understanding, flexibility and mutual respect between healthcare professionals and parents in paediatric care. Avoiding arrogance, promoting understanding and adapting to different situations improve collaboration. Professionals must be understanding and flexible regarding parents' unique circumstances, such as distance or caregiving responsibilities. These considerations align with family‐centred care concepts, emphasising the significance of respectful and collaborative relationships in improving patient and family experiences [9, 26, 39].

There are notable similarities between the earlier strategies and the existing literature on ‘good parents’ definitions. All highlight the significance of a humanistic approach that combines technical expertise with empathy and compassion [5, 7, 23, 27]. The emphasis on actively involving parents, fostering effective communication and supporting parental presence aligns with the state‐of‐the‐art definitions in North American studies.

Furthermore, previous studies emphasise the importance of providing emotional support, acknowledging parents' unique challenges and promoting shared decision‐making processes [2, 5, 24]. This last aspect may not be evident in our findings, as parental decision‐making is still a delicate issue in Brazil. Culturally, Brazilian parents depend on medical decisions and lack autonomy in their children's well‐being while hospitalised.

Fewer parents described how health professionals support them as good parents, which had broader but less frequent responses. This may reflect diverse and individualised experiences with healthcare teams, varying across institutions in Brazil and depending on parental autonomy in care. Each parent's experience is unique, and in the context of this research, it might reflect their personal journey and challenges when dealing with these professionals. The lack of insights on professional support may also highlight parents' limited reliance on or recognition of a collaborative care approach.

## 6 Limitations of the Study

Despite the contributions of this study, there are limitations to consider. The sample consisted of parents of seriously ill Brazilian children hospitalised in one hospital's PICU, which may limit the generalisability of the findings to other contexts or populations. However, one of the study's intentions was to explore cultural differences, as previous research has primarily been conducted in North America. Future research could examine the implications of these definitions and beliefs for parental well‐being, family dynamics and the development of support interventions tailored to the needs of Brazilian families with seriously ill children.

## 7 Implications and Recommendations for Practice

This study points to the finding that parents of critically ill children in Brazil construct their own ‘good parent’ identity based on activities such as being physically and emotionally present, being sheltered or safeguarded by parents, demonstrating emotion management and adequate communication with the clinical team. These results bring significant lessons to be learned here for paediatric critical care nurses that involve family‐centred practice behaviours that cut across clinical intervention, relational support and emotional care. Effective and empathetic communication, the active involvement of parents in care‐related decisions and the recognition of both their contribution and the emotional burden they carry are fundamental elements for nurses working in the critical care setting of the PICU. This study reaffirms that social contexts, interpersonal strategies used and individual experience influence what ‘good parent’ means to be dynamic rather than stable; they vary based on the context in which they function.

When PICU nurses seek to understand how each parent defines their role in their child's care, they are better equipped to build trusting relationships and deliver personalised and meaningful support.

Although this study did not adopt a specific theoretical model to describe parental beliefs, its findings provide a relevant practical foundation for developing approaches that centre the parents' perspective. Such approaches foster effective partnerships with families, particularly in settings of high clinical complexity, such as the PICU, where parental decision‐making is still emerging; nurses play a crucial role in engaging parents in the child's care within cultural and institutional boundaries. Our findings also highlight the importance of enhancing cultural sensitivity in caregiving, especially in complex scenarios shaped by Brazil's heterogeneous sociocultural contexts.

## 8 Conclusion

In conclusion, this study aimed to explore the beliefs of Brazilian parents regarding what it means to be a ‘good parent’ for seriously ill children in the PICU, as well as to examine the behaviours of healthcare professionals that support parents in fulfilling these roles. The findings provide valuable insights into the perspectives and definitions surrounding parental roles in the context of serious childhood illness.

The findings highlight the significance of parental presence, specific behavioural attributes and family unity. The emphasis on being physically and emotionally available, providing continuous support and actively participating in their child's care reflects the parents' commitment to ensuring their child's well‐being. Effective communication and providing accurate and timely information from healthcare professionals are crucial for parents to understand their child's condition and make informed decisions.

It is important to recognise the cultural context of the study, as these findings are specific to Brazilian parents. Cultural beliefs, values and societal norms significantly influence parental beliefs and behaviours in the context of serious childhood illness. Studying these topics in other cultures is essential for understanding the diversity of parental perspectives and developing culturally sensitive interventions.

This study contributes to the existing body of knowledge by shedding light on the beliefs and expectations of Brazilian parents in the PICU setting. The findings underscore the need for healthcare professionals and support services to consider the multifaceted needs of parents and tailor their approaches accordingly. Further research should enhance the understanding of the experiences and beliefs of parents from diverse cultural backgrounds. This will provide a comprehensive understanding of parental definitions and roles, and how these can shape their decision‐making process and overall care for seriously ill children, anchored in the professionals' awareness and support.

## Ethics Statement

Ethical approval was granted by the Institutional Review Board of Santa Casa de São Paulo Hospital, São Paulo, Brazil (5.339.610/December 2022).

## Consent

Written informed consent was obtained from all participants.

## Conflicts of Interest

The authors declare no conflicts of interest.

## Data Availability

The data that support the findings of this study are available from the corresponding author upon reasonable request.
